# Potential of the Virion-Associated Peptidoglycan Hydrolase HydH5 and Its Derivative Fusion Proteins in Milk Biopreservation

**DOI:** 10.1371/journal.pone.0054828

**Published:** 2013-01-24

**Authors:** Lorena Rodríguez-Rubio, Beatriz Martínez, David M. Donovan, Pilar García, Ana Rodríguez

**Affiliations:** 1 DairySafe Group, Department of Technology and Biotechnology of Dairy Products, Instituto de Productos Lácteos de Asturias (IPLA), Consejo Superior de Investigaciones Científicas (CSIC), Villaviciosa, Asturias, Spain; 2 Animal Biosciences and Biotechnology Laboratory, Animal and Natural Resources Institute, Beltsville Agricultural Research Center (BARC), Agricultural Research Service (ARS), USDA, Beltsville, Maryland, United States of America; Instituto Butantan, Brazil

## Abstract

Bacteriophage lytic enzymes have recently attracted considerable interest as novel antimicrobials against Gram-positive bacteria. In this work, antimicrobial activity in milk of HydH5 [a virion-associated peptidoglycan hydrolase (VAPGH) encoded by the *Staphylococcus aureus* bacteriophage vB_SauS-phiIPLA88], and three different fusion proteins created between HydH5 and lysostaphin has been assessed. The lytic activity of the five proteins (HydH5, HydH5Lyso, HydH5SH3b, CHAPSH3b and lysostaphin) was confirmed using commercial whole extended shelf-life milk (ESL) in challenge assays with 10^4^ CFU/mL of the strain *S. aureus* Sa9. HydH5, HydH5Lyso and HydH5SH3b (3.5 µM) kept the staphylococcal viable counts below the control cultures for 6 h at 37°C. The effect is apparent just 15 minutes after the addition of the lytic enzyme. Of note, lysostaphin and CHAPSH3b showed the highest staphylolytic protection as they were able to eradicate the initial staphylococcal challenge immediately or 15 min after addition, respectively, at lower concentration (1 µM) at 37°C. CHAPSH3b showed the same antistaphyloccal effect at room temperature (1.65 µM). No re-growth was observed for the remainder of the experiment (up to 6 h). CHAPSH3b activity (1.65 µM) was also assayed in raw (whole and skim) and pasteurized (whole and skim) milk. Pasteurization of milk clearly enhanced CHAPSH3b staphylolytic activity in both whole and skim milk at both temperatures. This effect was most dramatic at room temperature as this protein was able to reduce *S. aureus* viable counts to undetectable levels immediately after addition with no re-growth detected for the duration of the experiment (360 min). Furthermore, CHAPSH3b protein is known to be heat tolerant and retained some lytic activity after pasteurization treatment and after storage at 4°C for 3 days. These results might facilitate the use of the peptidoglycan hydrolase HydH5 and its derivative fusions, particularly CHAPSH3b, as biocontrol agents for controlling undesirable bacteria in dairy products.

## Introduction

*Staphylococcus aureus* is a bacterial pathogen responsible for a wide range of human and animal infections, including food poisoning caused by the ingestion of enterotoxins produced in food by enterotoxigenic strains [1], [2]. Staphylococcal enterotoxins are notoriously thermostable and maintain their stability even after the thermal treatments customarily utilized in the food industry. This represents a threat to consumers and makes necessary the control of staphylococcal contaminants to avoid the production of high risk levels of enterotoxins [3].

Humans and domestic animals are the primary reservoirs of *S. aureus*, as this microorganism colonizes mucous membranes and skin. Thus, food handlers and animals are usually the primary source of *S. aureus* contamination of food products of animal origin [4]. *S. aureus* is also an important etiological agent of mastitis in cattle, goats and sheep [5], with the mastitic udder being a source of contaminated milk and milk-derived dairy products, along with the dairy farm environment and processing facilities [6]. Although an important food safety concern, *S. aureus* mastitis is difficult to eradicate and constitutes a serious economic problem for dairy herd management [7]. Several antimicrobial treatments are available for clinical mastitis differing in the antimicrobial agent, route of application, duration, probability of cure or recurrence, and cost [8]. However, this problem remains unsolved in part due to the ability of *S. aureus* to invade and reside intracellularly [9], within mammary cells, thereby evading most antibiotics, but also because of the high frequency of antibiotic resistance among *S. aureus* strains [10], [11].

Bacteriophage endolysins have been proposed as antimicrobials to control Gram positive bacteria due to their ability to degrade the bacterial cell wall resulting in lysis of the pathogen [12]. This bactericidal activity has been successfully used to control antibiotic-resistant pathogenic bacteria in animal models [13]. For instance, the pneumococcal lysin Cpl-1 protected a mouse model against pneumococcal bacteraemia and colonization by intravenous administration and topical nasal treatment, respectively [14]. More recently, staphylococcal lysins have also been used against staphylococcal infections in mouse models. This is the case for endolysins MV-L [15], LysGH14 [16] or the chimeric lysin ClyS [17] that protected mice against lethal doses of methicillin-resistant *S. aureus* (MRSA) by intraperitoneal injections. The effectiveness shown by the staphylococcal phage K endolysin, LysK, CHAP domain construct and the CHAP domain construct from the phage K tail-associated muralytic enzyme to eliminate *S. aureus* from the nares of challenged mice and rats, respectively, supports the potential use of phage lytic proteins’ catalytic domains as antimicrobials [18], [19].

Lysostaphin is a well characterized peptidoglycan hydrolase produced by *Staphylococcus simulans* biovar. *staphylolyticus*. Its lytic action against *S. aureus* relies mainly on its N- terminal domain with glycylglycine endopeptidase activity that cleaves the pentaglycine cross bridges present in staphylococcal peptidoglycan, while its C- terminal domain promotes its specific binding to staphylococcal peptidoglycan [20]. It was shown to protect mammary glands against *S. aureus* challenge in both mice [21] and cattle [22]. It has also been shown that antimicrobial synergy exists *in vitro* between some phage endolysins and antibiotics or antimicrobial peptides of bacterial origin against *S. aureus* [18], [23], [24]. In this regard, the *in vitro* synergy observed between phage lytic proteins and lysostaphin was recently expanded to include the *in vivo* protection of murine mammary glands from an *S. aureus* challenge [25].

In addition to mammary gland protection, phage endolysins might also serve to inhibit undesirable bacterial growth for food biocontrol purposes [24]. The staphylococcal phage vB_SauS-phiIPLA88 endolysin, LysH5, has been demonstrated to control *S. aureus* growth in milk. The purified protein was able to rapidly kill *S. aureus* growing in pasteurized milk with a 10^6^ CFU/ml inoculum undetectable after 4 h of co-incubation with 1.6 µM LysH5 at 37°C [26].

In addition to endolysins, there is a largely untapped group of phage lytic proteins the virion-associated peptidoglycan hydrolases (VAPGHs) that are involved in local cell-wall degradation to facilitate the injection of phage DNA into the cell cytoplasm [27]. These PGHs have been reported to be encoded by phages infecting *S. aureus* [28], [29] and other bacterial species [30]–[33]. Their antimicrobial activity was first postulated in 1940, with ‘lysis from without’ that takes place when a very high number of phages are adsorbed onto the host cell [34].

In a previous work, we have described the VAPGH HydH5 encoded by the *S. aureus* phage vB_SauS-phiIPLA88. Bioinformatic analysis of the protein sequence revealed two putative domains, a cysteine, histidine-dependent amidohydrolase/peptidase (CHAP) domain [35], [36]; and a LYZ2 (lysozyme subfamily 2) domain [28], which conferred antimicrobial activity against *S. aureus* [37]. Three different fusion proteins obtained between lysostaphin and HydH5 (CHAPSH3b, HydH5SH3b and HydH5Lyso) showed significantly greater activity than the parental protein HydH5, and lysed both bovine and human *S. aureus*, including MRSA N315, and human *Staphylococcus epidermidis* strains [38].

In this work, we have assessed the antimicrobial ability of HydH5 and its derivative fusion proteins in milk, in order to explore new biopreservation strategies to effectively inhibit *S. aureus* growth in dairy products.

## Materials and Methods

### Bacterial Strains and Culture Conditions

*S. aureus* Sa9, isolated from a mastitic milk sample, was used as the indicator strain for lytic activity [39]. This bacterium was grown in TSB broth (Tryptic Soy Broth, Difco, Franklin Lakes, NJ, US) at 37°C for up to 18 h with vigorous shaking. For selective counting Baird–Parker agar supplemented with egg yolk tellurite (Scharlau Chemie, S.A. Barcelona, Spain) was used in commercial whole extended shelf life (ESL) (125°C, 4 sec) milk samples, and ChromoID *S. aureus* plates (Biomérieux, Marcy l’Etoile, France) in raw and pasteurized (72°C, 15 sec) (whole and skim) milk samples. *E. coli* BL21(DE3)/pLysS containing the pET21a-HydH5 and derivative plasmids were used to overproduce the lytic proteins HydH5, HydH5SH3b, HydH5Lyso and CHAPSH3b [38].

### Microbiological and Physicochemical Analyses of Milk

Microbiological and physicochemical analyses were performed in commercial cow’s whole ESL milk whole and skim raw milk (the latter was centrifuged at 6,000×*g* for 20 min to remove fat) and whole and skim pasteurized milk supplied by a collaborating farm. Samples of milk (500 ml) were aseptically sampled. Serial dilutions of milk were made in quarter-strength Ringer solution (Oxoid, Basingstoke, Hampshire, UK) and plated in duplicate on the appropriate agar medium. Total bacterial counts were performed in the different types of milk by deep-plating appropriate dilutions on Plate Count Agar (32°C, 72 h). *S. aureus* counting was performed as indicated above.

Total solids, fat and protein content were determined according to the International Dairy Federation [40]–[42].

### Protein Purification

Protein purification was performed as previously described [38]. Purity of each preparation was determined in 15% (w/v) SDS-PAGE gels. Electrophoresis was conducted in Tris–Glycine buffer at 20 mA for 1 h in the BioRad Mini-Protean gel apparatus. Protein was quantified by the Quick Start Bradford Protein Assay (BioRad, Hercules, CA). Quantification of lytic activity was performed by turbidity reduction assays against live *S. aureus* Sa9 cells prepared as previously described [43], [44].

### Challenge Tests in Milk

HydH5, HydH5SH3b or HydH5Lyso proteins (3.5 µM), CHAPSH3b protein (1 µM) and lysostaphin (1 µM) were individually added to 2 ml of whole ESL milk inoculated with 10^4^ CFU/ml of *S. aureus* Sa9 and incubated at 37°C for 6 h. CHAPSH3b (1.65 µM) was also assayed at room temperature (RT) for the same period. The anti-staphylococcal activity of CHAPSH3b (1.65 µM) was further assayed in whole and skim raw milk and in whole and skim pasteurized milk inoculated with 10^3^ CFU/ml at 37°C and RT for 2 h. Challenged milk without lytic protein additions were used as controls.

Samples were taken at different times throughout the incubation period and survival of *S. aureus* Sa9 was determined by serial dilution plating onto Baird-Parker plates for ESL milk samples (37°C, 48 h) and ChromoID *S. aureus* plates (37°C, 24 h) for raw and pasteurized milk samples, respectively. ChromoID *S. aureus* is a selective and differential culture medium for *Staphylococcus* sp, in which different staphylococcal species are distinguished by the colour of colonies (green for *S. aureus*; pink in *S. saprophyticus*; purple in *S. xylosus*; white in *S. epidermidis*). This chromogenic medium inhibits other Gram positive bacteria, Gram negative bacteria and yeasts.

### CHAPSH3b Fusion Protein Stability in Milk

To test the stability of CHAPSH3b in milk, 1.65 µM protein was added to 2 ml whole raw milk and kept at 4°C for 3 days. Samples (250 µL) were taken every day, challenged with 10^3^ CFU/ml of *S. aureus* Sa9 and incubated at RT for 15 min. Staphylococcal viable counts in the presence and in the absence of the antimicrobial protein were determined by serial dilution plating onto ChromoID *S. aureus* plates. Results were expressed as the percentage of viable counts reduction compared to the untreated control.

### CHAPSH3b Pasteurization Treatment

Commercial whole ESL milk and whole raw milk containing CHAPSH3b (1.97 µM) were pasteurized (72°C, 15 s) in a thermo cycler (BioRad Laboratories, Hercules, CA, USA). Samples were cooled at RT for 15 min, further inoculated with 10^3^ CFU/ml of *S. aureus* Sa9 and incubated for 0, 15, 30, 60 and 120 minutes at RT. Staphylococcal viable counts were determined as indicated above and results also expressed as the percentage reduction of viable counts.

### Statistical Analysis

Statistical analysis was performed using the SPSS-PC +11.0 software (SPSS, Chicago, IL, USA). Staphylococcal CFU data were subjected to one-way ANOVA within each sampling time. Types of anti-staphylococcal protein (HydH5, HydSH3b, HydH5lyso and CHAPSH3b) were compared against the untreated control. Data of cold storage stability of protein CHAPSH3b were compared with one-way ANOVA and the LSD test was used for a comparison of means at a level of significance *P*<0.05.

## Results

### Microbiological and Physicochemical Characteristics of Milk

Total viable bacterial counts were below the detection limit (<10 CFU/ml) in ESL milk, whereas about 7.08×10^4 ^CFU/ml and 3.89×10^1 ^CFU/ml were detected in raw and pasteurized milk, respectively. Viable counts were lower in both raw skim (1.90×10^3^ CFU/ml); and pasteurized skim milk (1.20×10^1^ CFU/ml). *S. aureus* counts were only detected in whole raw milk (2.84–4.0×10^1^ CFU/ml) and they kept below 10^2^ CFU/ml throughout 2 h of incubation.

Results of gross composition are shown in Table 1. Mean values of total solids, fat and protein contents of whole commercial ESL milk, and raw (whole and skim) and pasteurized (whole and skim) milk were within the standards of commercial and farmhouse milk.

**Table 1:** Gross composition of milk used in the HydH5 and its derivative fusion proteins antistaphyloccal assays^a^.

	ESL^b^	Whole		Skim	
		Raw	Pasteurized	Raw	Pasteurized
**Total solids** c	12.28±0.08	11.14±0.01	11.17±0.03	11.04±0.12	11.01±0,09
**Fat** d	3.54±0.02	3.18±0.03	3.20±0.05	0.1±0.02	0.15±0.04
**Protein** d	3.06±0.09	3.09±0.12	3.09±0.14	3.07±0.12	3.08±0.16

a Data reported are means ±standard deviations of two independent milk samples.

b ESL: extended shelf life milk.

c Total solids: data expressed as g/100 g milk.

d Fat/Protein: expressed as g/100 g milk.

### HydH5 and its Derivative Fusion Proteins have Antimicrobial Activity against *S. aureus* Sa9 in Commercial ESL Milk

**Figure 1:**
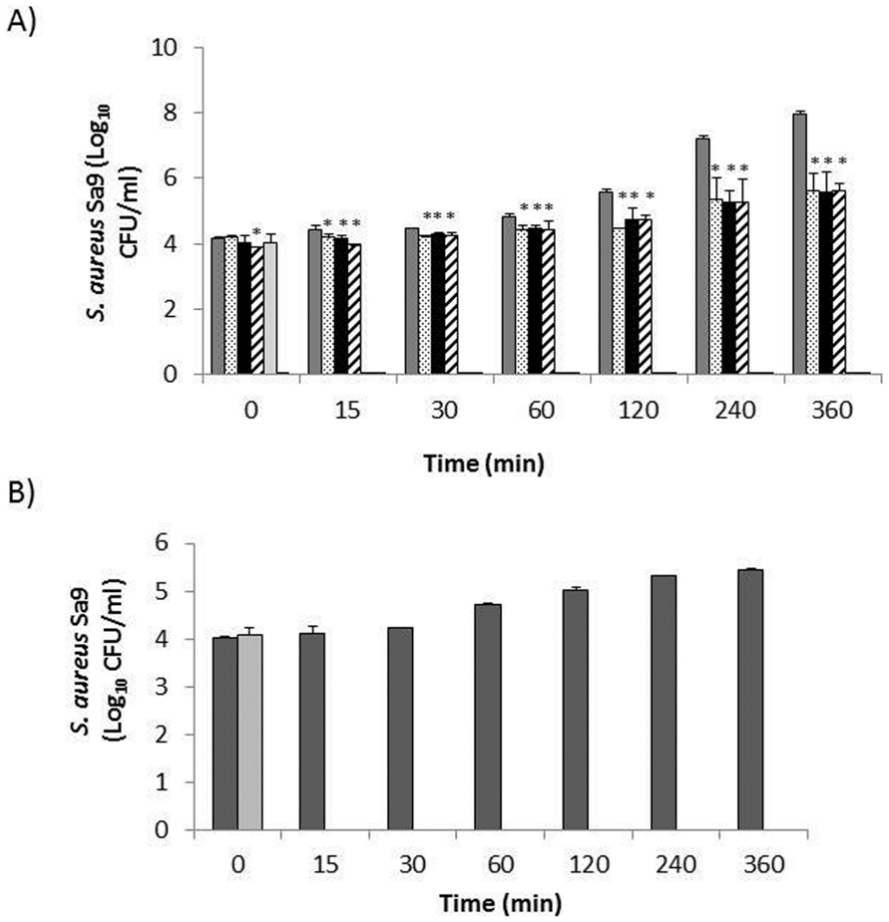
Antimicrobial activity of HydH5, lysostaphin, and its derivative fusion proteins in commercial whole ELS milk. Milk was inoculated with 10^4^ CFU/ml of *S. aureus* Sa9 and incubated for 0, 15, 30 min, 1, 2, 4 and 6 h at 37°C either without lytic protein (control; dark grey bars) or in the presence of : A) 1 µM CHAP-SH3b (light grey bars), and 3.5 µM HydH5 (stippled bars), 3.5 µM HydH5SH3b (black bars) and 3.5 µM HydH5Lyso (diagonal stripes bars); 1 µM lysostaphin (gross line on X axis); (B) 1.65 µM CHAPSH3b (light grey bars), control without protein (dark grey bars), at room temperature. Values expressed as log_10_ CFU/ml are the means ± standard deviations of two independent experiments. Bars having an asterisk are significantly different from the control (*P≤0.05). *S. aureus* detection threshold (<10 CFU/ml).

The antimicrobial activity of HydH5, its derivative fusion proteins and lysostaphin was assessed in commercial whole ESL milk inoculated with 10^4 ^CFU/ml of *S. aureus* Sa9. The effect of the different proteins on *S. aureus* growth was first tested at 37°C. In the absence of antimicrobial proteins (control cultures) the staphylococcal strain grew from 10^4^ to 6.5×10^4^ CFU/ml during the first hour of incubation with a more robust increase in CFU subsequently, achieving 8.9×10^7^ CFU/ml at the end of six hours (Fig. 1A). The addition of HydH5, HydH5SH3b and HydH5Lyso (3.5 µM) to the *S. aureus* inoculated milk resulted in an immediate effect on *S. aureus* viability, with the viable counts maintained below the time zero control counts (immediately after addition of the antimicrobials). At time 0, only the viable counts in HydH5Lyso treated cultures were significantly different (*P*<0.05) compared to the control cultures. From 15 min onwards, the inhibitory effect of each of the proteins on *S. aureus* viability was significant (*P*<0.01 at 15 min and *P*<0.001 thereafter). The greatest reduction in viable counts (about 2.34±0.01 log CFU/ml) was detected at the end of the 6 h incubation period (Fig. 1A). These activities are, however, far from lysostaphin antistaphylococcal activity since 1 µM of this bacterial peptide resulted in an immediately kill of the *S. aureus* population and no viable counts were detected even at time 0. In addition, no-re-growth was observed afterwards (data not shown). Only the fusion protein CHAPSH3b showed an inhibitory effect on *S. aureus* similar to lysostaphin since 1 µM resulted in a complete clearance of the pathogen 15 min after addition without further re-growth throughout the assay period (6 h) (Fig. 1A). Likewise, viable counts became undetectable immediately after the addition of 1.65 µM CHAPSH3b (data not shown). At RT, the inhibitory effect of CHAPSH3b decreased slightly with a higher protein concentration (1.65 µM) required to fully eliminate *S. aureus* in 15 min after addition (Fig. 1B), whereas a continuous proliferation of the staphylococcal population occurred in the control cultures.

### CHAPSH3b Fusion Protein is Effective in Raw Milk and Highly Effective in Pasteurized Milk

**Figure 2:**
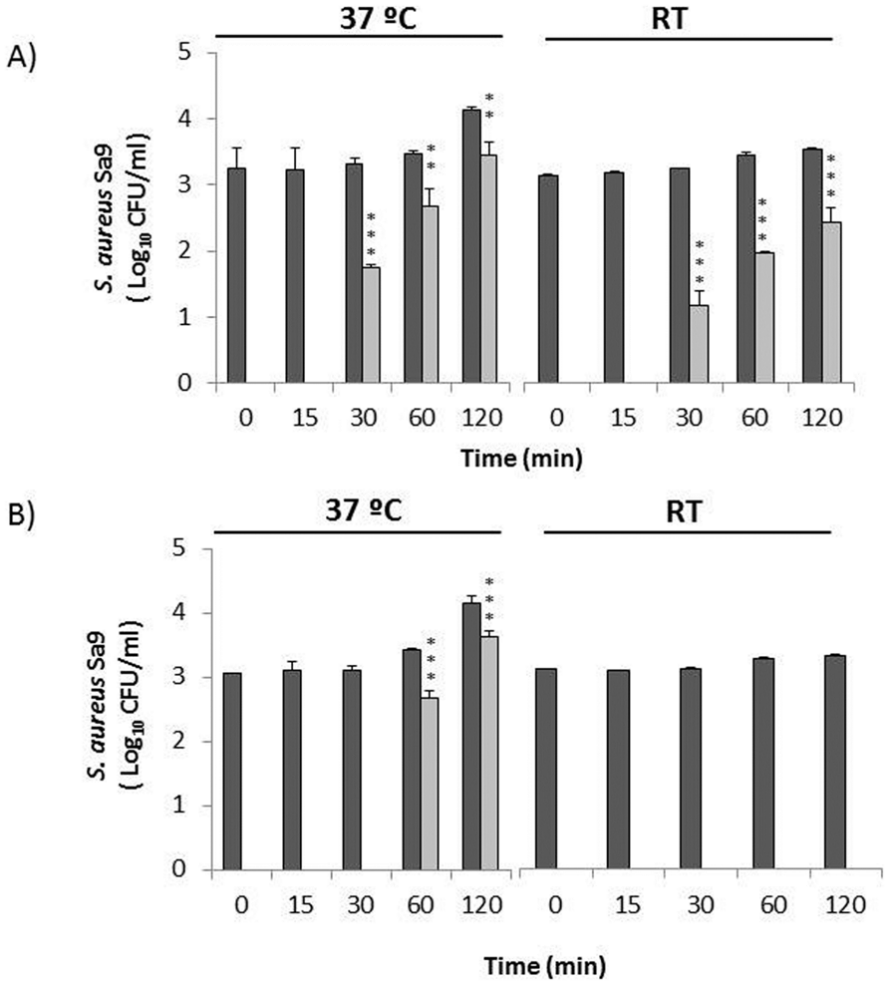
Antimicrobial activity of CHAPSH3b fusion protein in whole milk. Milk was inoculated with 10^3^ CFU/ml of *S. aureus* Sa9 and incubated in the presence of 1.65 µM CHAPSH3b for 0, 15, 30, 60 and 120 min either at RT or 37°C in: A) raw milk and B) pasteurized milk. Dark grey bars indicate *S. aureus* Sa9 control culture and light grey bars *S. aureus* Sa9+ CHAPSH3b. Values, expressed as log CFU/ml, are the means ± standard deviations of two independent experiments. Bars having asterisks are significantly different from the control (**P<0.01; ***P<0.001). *S. aureus* detection threshold (<10 CFU/ml).

**Figure 3:**
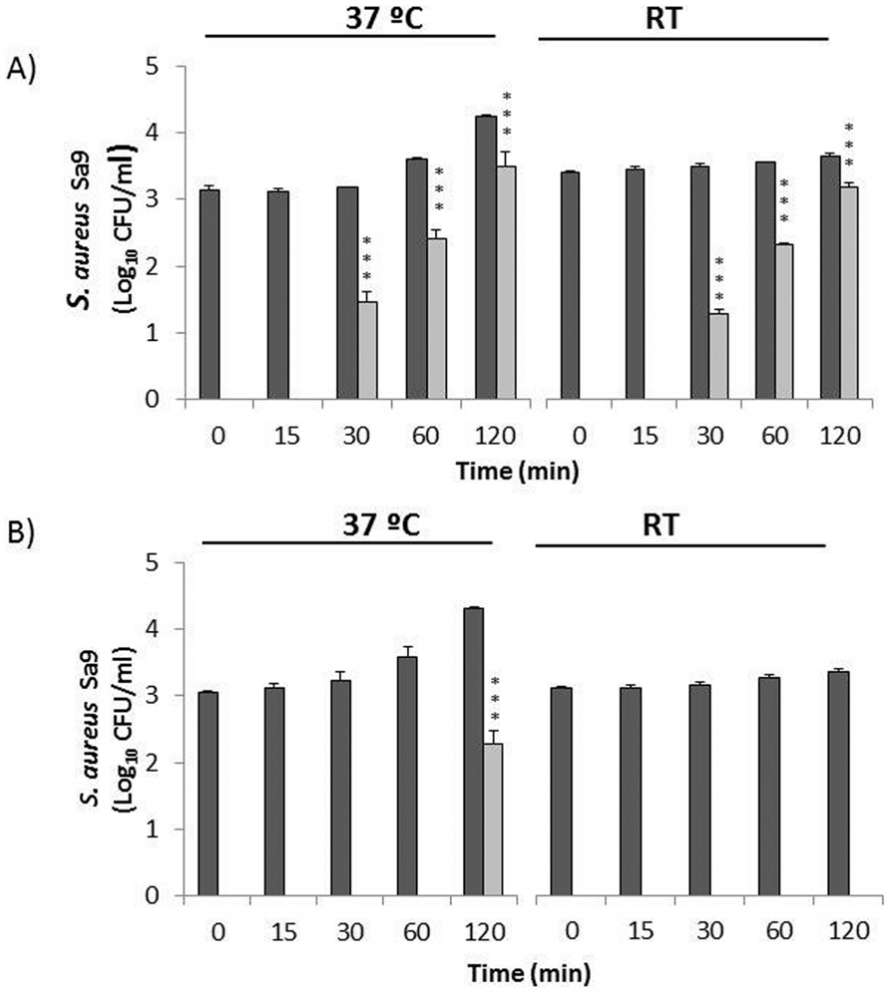
Antimicrobial activity of CHAPSH3b protein in skim milk. Milk was inoculated with 10^3^ CFU/ml of *S. aureus* Sa9 and incubated in the presence of 1.65 µM CHAPSH3b for 0, 15, 30, 60 and 120 min either at RT or 37°C in: A) raw milk and B) pasteurized milk. Dark grey bars indicate *S. aureus* Sa9 control culture and light grey bars *S. aureus* Sa9+ CHAPSH3b. Values, expressed as log CFU/ml, are the means ± standard deviations of two independent experiments. Bars having asterisks are significantly different from the control (***P<0.001). *S. aureus* detection threshold (<10 CFU/ml).

The effect of the CHAPSH3b protein was tested against *S. aureus* Sa9 strain (10^3^ CFU/ml) in whole and skim raw milk at both 37°C and RT. As shown in Figure 2A, the staphylococcal growth observed in the whole raw milk control cultures was immediately inhibited by addition of 1.65 µM of CHAPSH3b as no viable counts were detected at 37°C or RT and re-growth was prevented for ∼30 min of incubation. Thereafter, *S*. *aureus* growth was observed at both temperatures but CHAPSH3b treatment kept viable counts below the control counts throughout the 2 h experiment. CHAPSH3b showed higher growth inhibition at RT than at 37°C (Fig. 2A). Significant differences between control and treated cultures were observed throughout the remaining 2 h incubation period at both RT (*P*<0.001) and 37°C (*P*<0.001 at 30 min and *P*<0.01 at 60 and 120 min of sampling time). At the end of the incubation period, the presence of the antimicrobial protein resulted in a reduction of 1.09±0.12 and 0.7±0.17 log CFU/ml at RT and 37°C, respectively, compared to the control cultures. The level of indigenous *S. aureus* in raw milk was also monitored through the incubation period as an additional control. This population remained below 10^2^ CFU/ml and was also sensitive to CHAPSH3b (data not shown).Similar staphylococcal growth kinetics was observed in skim raw milk in the presence of CHAPSH3b (Fig. 3A). As in whole milk (Fig. 2), re-growth also occurred after 30 min and the antimicrobial protein exhibited higher inhibitory activity at RT. Differences in staphylococcal viable counts between VAPGH-treated and control samples were significant (*P*<0.001) at both 37°C and RT. The final reduction in staphylococcal CFU was similar at 37°C (0.75±0.23 log CFU/ml) and lower (0.41±0.09 log CFU/ml) at RT than in whole raw milk (Fig. 3A).

CHAPSH3b was more effective in reducing *S. aureus* Sa9 in pasteurized milk (whole and skim) as is shown in Figure 2B and 3B. At RT, CHAPSH3b (1.65 µM) was able to reduce *S. aureus* viable counts to undetectable levels in whole and skim milk immediately after addition, and no re-growth was detected for 2 h thereafter. At 37°C, the presence of CHAPSH3b prevented staphylococcal re-growth for over 30 min in whole milk and for more than 1 h in skim milk. At the end of the incubation period, the final staphylococcal population in whole and skim pasteurized milk was 0.75±0.23 (*P*<0.001) and 2.02±0.21 log CFU/ml (*P*<0.001) lower than the control, respectively, in the presence of CHAPSH3b. In order to rule out the possibility of CHAPSH3b-resistant colonies confounding the data, ten *S. aureus* colonies were randomly selected from the selective agar plates used for viable count determination. Turbidity reduction assays of each colony performed in the presence of 1 µM of CHAPSH3b indicate that all were sensitive to lysis by CHAPSH3b to the same extent as the inoculated strain (data not shown).

### CHAPSH3b Remains Active after Storage at 4°C in Milk and after Pasteurization Treatment

**Figure 4:**
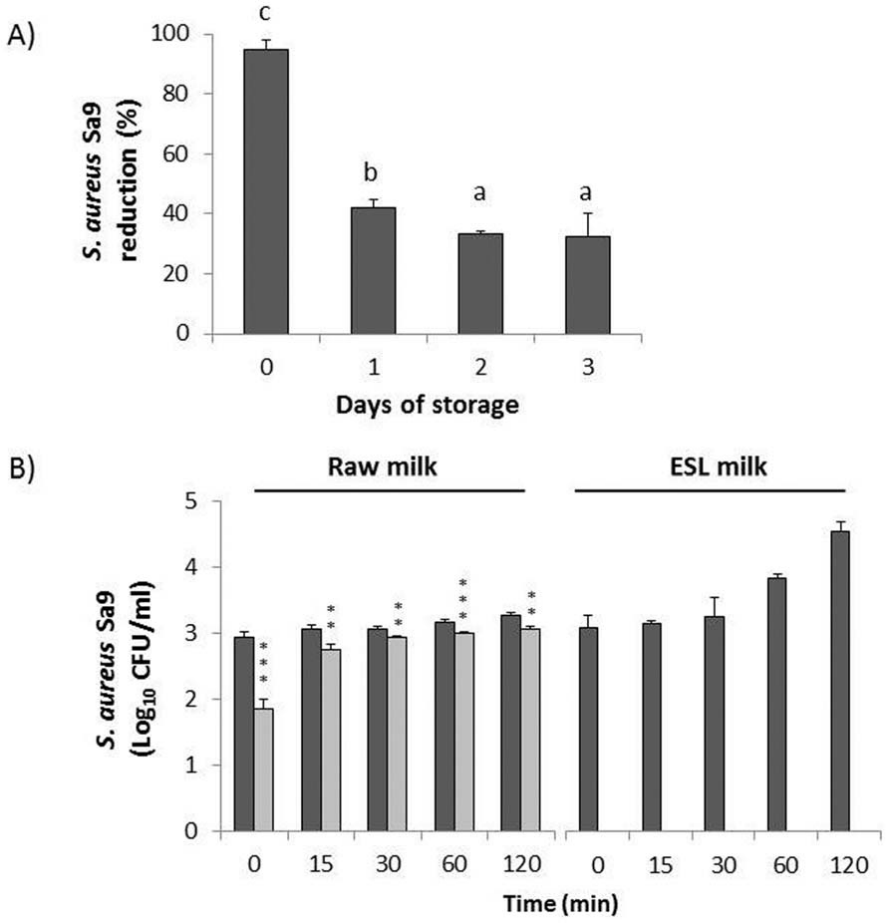
Cold storage stability and pasteurization resistance of CHAPSH3b in milk. A) 1.65 µM CHAPSH3b was stored in raw milk at 4°C for 3 days. Samples were taken every day, inoculated with 10^3^ CFU/ml of *S. aureus* Sa9 and incubated for 15 min at room temperature before plating. Non-cold storage protein was used as control. Cold storage stability was expressed as the percentage reduction of *S. aureus* Sa9 CFU/ml after CHAPSH3b addition. Values are the means ± standard deviations of two independent experiments. Bars having different letters are significantly different (*P*<0.05). B) 1.97 µM CHAPSH3b was pasteurized at 72°C for 15 s in raw milk (left) and commercial pasteurized milk (right). Samples were inoculated with 10^3^ CFU/ml and incubated for 0**,** 15, 30, 60 and 120 min at room temperature before plating. *S. aureus* inoculated cultures without lytic protein addition were used as control (dark grey bars). Light grey bars indicate *S. aureus* Sa9+ CHAPSH3b. Data from pasteurized samples with CHAPSH3b activity was expressed as log CFU/ml. Values are the means ± standard deviations of two independent experiments. Bars having asterisks are significantly different from the control (**P<0.01; ***P<0.001).

To assess the CHAPSH3b stability in milk, challenge assays were performed after storage of the protein (1.65 µM) in refrigerated raw milk for 3 days. As shown in Figure 4A, the non cold-stored protein reduced the initial staphylococcal population (10^3^ CFU/ml) by 94% after just 15 min at RT. The inhibitory activity of CHAPSH3b decreased significantly (*P*<0.05) with the cold storage time as compared with the non-cold stored protein but the remaining activity was still able to kill 42%, 33% and 32% of the *S. aureus* population following storage in milk at 4°C for one, two and three days, respectively. No significant differences in the anti-staphylococcal activity after 2 and 3 days of cold storage were detected (*P*>0.05). To test the stability of CHAPSH3b under high temperature treatment, the protein (2 µM) was subjected at pasteurization (72°C, 15 s) in both commercial ESL whole milk and raw whole milk and further challenged with *S. aureus* at RT. Pasteurization in ELS milk did not affect the inhibitory activity of CHAPSH3b since no viable counts were detected in treated cultures throughout the incubation period (Fig. 4B). However, the heat treatment in raw milk clearly reduced CHAPSH3b activity as only partial inhibition of *S. aureus* CFUs was observed in the first 15 min of treatment (Fig. 4B) compared to the undetectable CFUs that was observed in untreated raw milk cultures spiked with a lower concentration (1.65 µM) of unpasteurized protein (Fig. 2A). Nevertheless, the reductions in viable counts between control and treated cultures were significant throughout the incubation period *(P*<0.001 at time 0 and 60 min; *P*<0.01 at time 15, 30 and 120 min).

## Discussion

Current food safety depends on a combination of preventive hygiene-based approaches that are focused on minimizing the microbial contamination of raw material that mainly include physical and chemical decontamination treatments aimed to remove the microbial contamination in food products [45]. Due to the increasing consumer demand for natural, nutritious and fresh-tasting foods, the food industry is interested in replacing traditional preservation techniques (e.g. heat and chemical treatments) whenever possible, to avoid the risk of sensory quality changes or the presence of unwanted chemical residues in foods [46]. Food preservation treatments based on natural antimicrobials such as bacteriocins, bacteriophages or phage-derived lytic enzymes could help to fight against pathogenic and spoilage bacteria along the food chain and are not expected to alter the sensory change or other undesirable effects of traditional treatments [24]. The application of bacteriocins in food safety has been widely studied for the last two decades [47], but food biopreservation based on phages and phage-derived lytic enzymes is a more recent avenue of research [48]. So far, phage derived lysins have been mainly assayed in veterinary and human medical model approaches [12], [49], and less attention has been paid to their potential role as food biopreservatives [50]. Nevertheless, some phage lytic enzymes have shown antibacterial activity in milk. This is true for *S. aureus* bacteriophage vB_SauS-phiIPLA88 endolysin LysH5 that completely inhibited *S. aureus* growth in commercial pasteurized milk after 4 h of treatment [26], or the fusion proteins λSA2-E-Lyso-SH3b (streptococcal λSA2 endolysin endopeptidase domain fused to the lysostaphin SH3b domain) and λSA2-E-LysK-SH3b (streptococcal λSA2 endolysin endopeptidase domain fused to the staphylococcal phage K endolysin SH3b domain) that showed anti-staphylococcal activity in ultra-high temperature (UHT) milk by reducing the bacterial load by 3 and 1 log CFU/ml, respectively, within 3 h of incubation [51]. Recently, it has been also reported that *Listeria* bacteriophage endolysin LysZ5 was able to kill 4 log CFU/ml of *L. monocytogenes* within 3 h at 4°C in soya milk [52].

The peptidoglycan hydrolase HydH5 encoded by the *S. aureus* phage vB_SauS-phiIPLA88 [37] and the fusion proteins between HydH5 and lysostaphin (CHAPSH3b, HydH5SH3b and HydH5Lyso) have all been shown to yield staphylolytic activity in zymogram, plate lysis and turbidity reduction assays [38]. In this work, these constructs have been assessed as antimicrobial additives for preventing the growth of *S. aureus* in milk. HydH5, HydH5SH3b and HydH5Lyso showed staphylolytic activity in commercial whole ESL milk but were clearly less effective than lysostaphin and CHAPSH3b activities. Lysostaphin and CHAPSH3b (1 µM) were able to reduce the *S. aureus* load by 4-log CFU/ml immediately or 15 min after addition at 37°C, respectively, while nearly four times as much (3.5 µM) of the other constructs were needed to obtain just a reduction of the staphylococcal counts throughout the incubation period compared to the untreated cultures.

These findings are consistent with our previous results. In fact, the CHAP domain of HydH5 when fused to the lysostaphin SH3b domain showed a 4.8-fold higher activity, compared to full length HydH5 [38]. The high activity shown by CHAPSH3b in ESL milk, prompted us to broaden the assays on milk with a broader range of treatments. Accordingly, CHAPSH3b activity was assessed in raw (whole and skim) and pasteurized (whole and skim) milk.

CHAPSH3b is active in whole and skim raw milk at 37°C and RT, as the protein was able to reduce 10^3^ CFU/ml below the detection limit (<10 CFU/ml) for 30 min. The staphylolytic activity, however, was lower than in high-heat treated milk yielding less of a reduction in viable counts, despite a higher concentration of enzyme (1.65 µM *versus* 1 µM). Of note, the indigenous *S. aureus* contamination of raw milk do not seem to have interfered in the CHAPSH3b activity because it was shown to be sensitive and hardly accounts for the total *S. aureus* population once Sa9 was added. Pasteurization of milk clearly enhanced CHAPSH3b staphylolytic activity in both whole and skim milk at both temperatures. Apparently, something in the raw milk is hampering the CHAPSH3b activity. One possibility is heat-sensitive components such as immunoglobulin M and agglutinins in so far as they have been reported to promote the formation of cell clumps [53] that would likely make it more difficult for the antibacterial protein to reach the staphylococcal cells sequestered inside the clumps. These components of raw milk have also been previously reported to hamper phage adsorption [54]. In contrast, CHAPSH3b activity does not seem to be affected by fat globules in milk since similar kinetics of staphylococcal inhibition were observed in whole or skim milk despite the fact that bacterial clumps have also been associated with fat globules [55].

Although structural and chemical composition of food can negatively affect the ability of antimicrobials to reach the pathogen [56], the addition of CHAPSH3b to different types of milk yielded an immediate reduction in *S. aureus* viable counts with the only exception being heat-treated protein in raw milk. This suggests a quick reaction to CHAPSH3b which is reminiscent with previous data with *Listeria monocytogenes* phage endolysins Ply118 and Ply500. The cell binding domain of these proteins showed a rapid and saturation-dependent binding to *L. monocytogenes* cell surface within 15 s, with no further increase [57]. A high affinity for staphylococcal cells, especially MRSA, was also described for the endolysin LysGH15 [17]. The staphylococcal re-growth observed at latter sampling times could be attributed to those cells that were sequestered and did not see the CHAPSH3b at the beginning of the treatment. However, it should be noted that the number of bacteria attained by the end of the assay period (clearly below the critical threshold of 10^5^ CFU/ml for production of hazardous enterotoxins levels to consumers) does not present a high-risk of enterotoxin contamination of milk [58]. In addition, no CHAPSH3b resistant bacteria were isolated from lysin-treated milk. Therefore, insensitivity to CHAPSH3b appears to be a rare event under the experimental conditions tested. Other researchers also failed to detect resistance against endolysins used to control the growth of Gram positive bacteria, such as *Bacillus anthracis* [59] and *Streptococcus pneumoniae* [14].

Of note, CHAPSH3b showed higher activity at RT than at 37°C in raw and pasteurized milk. The lower growth rate of *S. aureus* at RT could account for the higher effectiveness of CHAPSH3b. The demonstrated 3 day longevity of CHAPSH3b in cold milk supports the notion of using CHAPSH3b as a potential staphylolytic agent to prevent *S. aureus* development during an unexpected breakdown in cold storage and thus, enhance food safety.

The ability of CHAPSH3b (1.65 µM) to kill up to 10^3^ CFU/ml in raw milk in the first 30 min of treatment at 37°C along with its proven activity against MRSA strains [38] points to CHAPSH3b as a potential candidate to control *S. aureus* infections in cows’ mammary glands. Previous reports have shown the effectiveness of chimeric phage lysins to kill mastitis-causing *S. aureus* in murine mammary glands [25]. The safety of endolysins has also been determined since experimental mice to which endolysin was administered did not exhibit adverse physiological effects [60].

The stability of CHAPSH3b after exposure at high temperatures (72°C, 15 s) and cold storage in milk has a clear technical interest for dairy products protection since this staphylolytic protein could be added to raw milk before thermal processing to control any potential contamination by *S. aureus*. CHAPSH3b thermostability is consistent with our previous results which showed that HydH5 retained activity after heat treatment (5 min treatment at 100°C) [37]. Recently, *Listeria* bacteriophage peptidoglycan hydrolases also revealed a high thermostability retaining up to 35% activity after 30 min of incubation at 90°C [51]. By contrast, the lytic activity of some phage endolysins was destroyed by heat treatment [26], [61]. However, none of these assays were performed in milk, work that is sorely needed.

Regarding the lytic proteins’ stability in cold storage, prior to this study, the existing data were obtained in aqueous solutions but not in milk. This is the case for CHAP_k_ that retained up to 70% of its lytic activity after being stored at 4°C for one month [18]. By contrast, the remaining lytic activity of CHAPSH3b was ∼33% after 3 days of storage a 4°C in raw milk. As indicated above, the reduction of lytic activity in raw milk could be due to the presence of heat-sensitive components in raw milk that hamper the access of the lytic protein to its target on the cell wall of the bacterial host [56]. Increasing the concentration of the lytic protein might enable us to overcome this limited activity in raw milk. Indeed, 3.30 µM (100 µg/ml) CHAPSH3b was able to kill 10^3^ CFU/ml of *S. aureus* in raw milk and no re-growth was observed within 2 h (data not shown).

Our findings demonstrate the ability of HydH5-derived proteins to inhibit the development of *S. aureus* in milk, with CHAPSH3b being particularly effective. The high anti-staphylococcal activity of CHAPSH3b along with its thermostability might enable this protein to be applied directly to raw milk after milking. Overall, our results suggest that phage lytic proteins might be useful as a valuable hurdle to prevent *S. aureus* growth in milk and presumably in other dairy products.
